# Is ChatGPT’s Knowledge and Interpretative Ability Comparable to First Professional MBBS (Bachelor of Medicine, Bachelor of Surgery) Students of India in Taking a Medical Biochemistry Examination?

**DOI:** 10.7759/cureus.47329

**Published:** 2023-10-19

**Authors:** Abhra Ghosh, Nandita Maini Jindal, Vikram K Gupta, Ekta Bansal, Navjot Kaur Bajwa, Abhishek Sett

**Affiliations:** 1 Biochemistry, Dayanand Medical College and Hospital, Ludhiana, IND; 2 Community Medicine, Dayanand Medical College, Ludhiana, IND; 3 Healthcare, Deloitte Consulting US India Pvt Ltd, Bangalore, IND

**Keywords:** ai-based education, medical biochemistry, medical education, artificial intelligence, chatgpt

## Abstract

Introduction

ChatGPT is a large language model (LLM)-based chatbot that uses natural language processing to create humanlike conversational dialogue. It has created a significant impact on the entire global landscape, especially in sectors like finance and banking, e-commerce, education, legal, human resources (HR), and recruitment since its inception. There have been multiple ongoing controversies regarding the seamless integration of ChatGPT with the healthcare system because of its factual accuracy, lack of experience, lack of clarity, expertise, and above all, lack of empathy. Our study seeks to compare ChatGPT’s knowledge and interpretative abilities with those of first-year medical students in India in the subject of medical biochemistry.

Materials and methods

A total of 79 questions (40 multiple choice questions and 39 subjective questions) of medical biochemistry were set for Phase 1, block II term examination. Chat GPT was enrolled as the 101st student in the class. The questions were entered into ChatGPT’s interface and responses were noted. The response time for the multiple-choice questions (MCQs) asked was also noted. The answers given by ChatGPT and 100 students of the class were checked by two subject experts, and marks were given according to the quality of answers. Marks obtained by the AI chatbot were compared with the marks obtained by the students.

Results

ChatGPT scored 140 marks out of 200 and outperformed almost all the students and ranked fifth in the class. It scored very well in information-based MCQs (92%) and descriptive logical reasoning (80%), whereas performed poorly in descriptive clinical scenario-based questions (52%). In terms of time taken to respond to the MCQs, it took significantly more time to answer logical reasoning MCQs than simple information-based MCQs (3.10±0.882 sec vs. 2.02±0.477 sec, p<0.005).

Conclusions

ChatGPT was able to outperform almost all the students in the subject of medical biochemistry. If the ethical issues are dealt with efficiently, these LLMs have a huge potential to be used in teaching and learning methods of modern medicine by students successfully.

## Introduction

Proper constructive language is one of the many things that makes the human race the smartest creature in this universe. In this technology-driven 21st century, we are trying to make instruments capable of generating constructive languages that can interact with humans seamlessly. The most successful field in this work is the development of artificial intelligence (AI). It uses computer algorithms supplemented by logic, statistics, linguistics, decision theory, cognitive psychology, cybernetics, and others involving minimal human intervention [1]. AI applications produce highly accurate classification models, regardless of the input data type by using different sensors, speech or image recognition, and accessing the vast information available on the World Wide Web [2]. The use of AI is rapidly increasing in every sector of our daily lives be it marketing, social networking, customer surveying, healthcare, and others and soon it will be capable of performing tasks with comparable intellectuality of a human brain [3-5].

During the last few months, an emerging sensation based on an AI platform called ChatGPT (https://chat.openai.com) has come up and gained significant attention among the public. Chat GPT is a chatbot, released by OpenAI that debuted in November 2022. It is a language model based on a deep learning architecture known as a generative pre-trained transformer (GPT) [6]. The model was first released in 2018 as GPT-1 and was trained in 117 million unique parameters back then and was able to produce human-like responses in natural conversations. In 2019, GPT-2 was released having 1.5 billion parameters and the latest GPT-3 having 175 billion parameters was released in 2020. Since then, it has become the most sophisticated AI language tool that can generate human-like conversations utilizing natural language depending on the inputs. The recent model of ChatGPT is powered by GPT 3.5, which is free to all users. A more developed version GPT-4 is also available for subscription, which is more collaborative and innovative, and can process image-based conversation as well [7].

ChatGPT is an LLM that can respond based on self-attention mechanisms, pre-training on a large corpus of internet-based text, and subsequent fine-tuning for specific tasks. It is a server-contained language model that is unable to browse or perform internet searches after receiving the query [8]. This makes the chatbot a different entity from conventional search engines (Google, Bing, Yahoo), as it can produce a definite answer based on the abstract relationship between words in the neural network.

Since the release of ChatGPT in November 2022, there has been a wave of proposals both supporting and opposing the integration of generative AI in the healthcare system. Medical knowledge is a constantly evolving subject, and health practitioners as well as medical students must study hard to keep up with the developments in the health sector. Medical students also need to learn to deal with various knowledge sources and their correctness at an early stage of their curricula as these techniques form the basis for clinical decisions that must be made in real-life clinical scenarios. As ChatGPT has access to information rather than deep knowledge, its responses to a wide spectrum of questions seem to generate factual knowledge, which sometimes lacks actual correlation with the given clinical scenario. As an AI chatbot like ChatGPT makes access to factual knowledge interactive in a mere smartphone, medical students can be expected to use this kind of service for their day-to-day use. However, the cause of concern for educators and teachers at medical school is that the responses generated by ChatGPT are still not widely validated and reliable, and exposure of medical students to these can cause issues in the quality of learning [9].

In 2019, the National Medical Commission of India revamped the undergraduate medical program and introduced a competency-based curriculum. The new program has made Bachelor of Medicine, Bachelor of Surgery (MBBS) more clinically oriented, and students are now frequently exposed to clinical and logical reasoning-type questions in their professional examinations [10].

After a vigorous search in the literature, to the best of our knowledge, no study has been found that can throw some insights on the comparison of knowledge and interpretative ability of ChatGPT with the performance of first professional MBBS students in India in the subject of biochemistry. So, this study was planned to evaluate and compare the scores and quality of answers generated by ChatGPT with those of first-year medical students of Dayanand Medical College and Hospital, Ludhiana in the subject of medical biochemistry.

Objectives

The study aimed to compare the knowledge and interpretative ability of ChatGPT with those of first-year MBBS students learning medical biochemistry. Specifically, the following objectives were investigated: a) comparison of scores of ChatGPT to different types of questions such as multiple-choice questions (MCQs), clinical reasoning, comprehensive questions, logical reasoning, and ethical reasoning with those of medical students as evaluated by the subject experts, b) relation of response time of ChatGPT with two different types (information-based vs. logical reasoning) of MCQs, and c) acceptability of answers given by ChatGPT and analysis of its weaknesses and strengths.

## Materials and methods

Study settings

**Figure 1:**
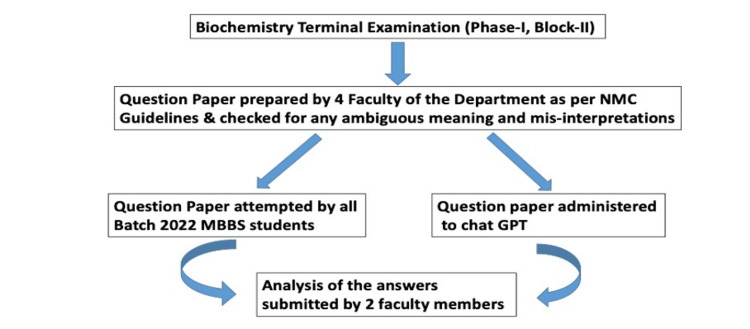
Flow diagram of our study design

The study was done collaboratively by the Department of Biochemistry and the Department of Community Medicine. Our institute enrolls 100 students for first-year MBBS. ChatGPT was considered as the 101st student to make it a real examination scenario. Syllabus of block I and block II were included in the examination. A total of 79 questions on the subject of medical biochemistry were prepared by the four faculties of the Department of Biochemistry for the second-term examination of the first-year students (Figure 8-13 of Appendix). Questions were made as per the undergraduate curriculum guidelines provided by the Undergraduate Medical Education Board of the National Medical Commission (Figure 1) [10]. The question sets were sent to Dean Academics of the institute and the affiliating university for checking of ambiguity and validation as a standard protocol, two weeks before the scheduled date of term examination.

Data collection

The term examination of MBBS students was held in the third week of June 2023. We started capturing the answers of ChatGPT during that same week. The free version of ChatGPT (based on GPT 3.5) developed by OpenAI was used and a free user account was created using email and logged in to the account. As ChatGPT has almost 15% user base in the USA only, we avoided the high traffic time according to USA time zones and selected the morning hours from 10 am to 12 noon IST for getting our answers [11]. Questions were copied from a Word document into the chat box of ChatGPT to get the answers. The first answer generated by the chatbot was taken as the final answer and the regenerate answer option was avoided. Every question was entered in a fresh session to avoid any retention bias. The time from pressing the enter key to the completion of the generation of the answer was also noted in the case of all MCQs with the help of an automated stopwatch. All the answers generated by ChatGPT were copied and pasted into a Word document for further processing. To maintain anonymity and avoid any bias, the answers generated by the chatbot were handwritten by a BSc Medical Lab Technology first-year student at our institute in the identical answer sheet used in the term examination of MBBS students. All the answer sheets, which included 100 copies of MBBS students and one copy of the chatbot, were then checked by two different subject experts in biochemistry. All the roll numbers written in the copies were masked. This helped us to remove all the identifiers, which could have created a bias during checking. Marks given by both the subject experts were tabulated and the mean was taken as the final mark for every question. The final marks were entered in the Excel sheet for further statistical analysis. A brief outline of the study is shown in Figure 1. 

Ethical statement

This study did not involve any human subjects. It only analyses the results of an educational examination that is routinely conducted in a teaching medical school. Therefore, a waiver was taken from the institutional ethics committee for the conduct of the study.

Statistical analysis

It was performed using Microsoft Excel 2019 version (Microsoft Corporation, Redmond, USA) and Jamovi software [12]. Statistical parameters like mean, standard deviation, median, and range were calculated. Independent student's t-test was applied for statistical comparison. Q-Q plot analysis was also done to check the distribution of homogeneity of data. 

## Results

**Figure 2:**
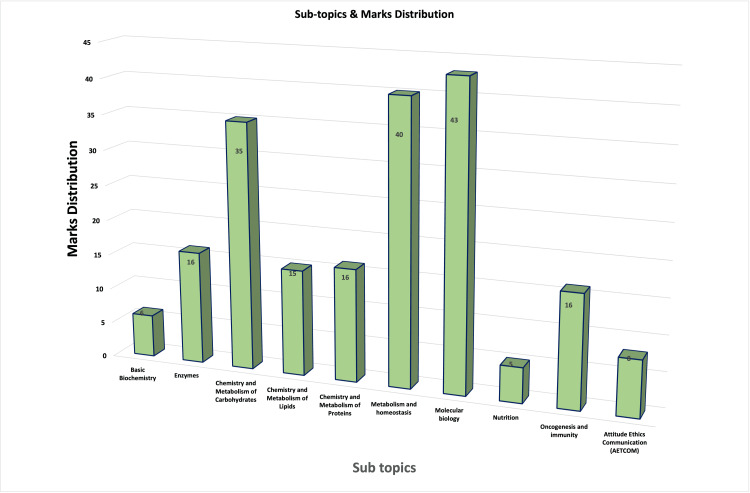
Subtopics and marks distribution of the examination

The AI chatbot answered a total of 79 questions, out of which 39 were subjective types and 40 were MCQs. Figure 2 shows the subject subtopics and distribution of marks in this knowledge-based assessment in biochemistry.

The question paper contained different types of questions. Table 1 shows the details of the type of questions and the marks allotted to each section.

**Table 1:** Type of questions and marks distribution in knowledge-based assessment of biochemistry

Biochemistry terminal examination (phase-I, block-II)	Number of questions	Marks
Clinical questions	9	25
Logical reasoning	9	32
Comprehensive	19	95
Ethical reasoning	2	8
MCQ (information-based)	25	25
MCQ (logical reasoning)	15	15
Total	79	200

As 40 MCQs had only a single answer, only single assessor checked these. The rest of the 39 questions (non-MCQ) were assessed by two different faculty assessors independently. There was excellent agreement between the two assessors (inter reliability with interclass coefficient being excellent, i.e., above 80%).

**Figure 3:**
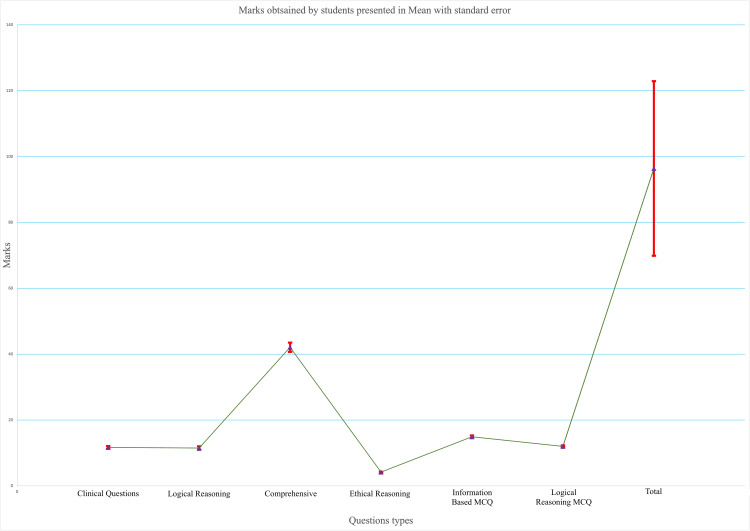
Descriptive analysis of students’ performance (n=100) Marks obtained are presented in mean with standard error

After evaluation of all the data, it was found that only 46 students were able to pass the exam (a student is considered to pass on obtaining at least 50% of the total marks) as indicated by the overall mean score of marks of students, which was below 100. The mean performance of students was above 50% in ethical reasoning and information-based MCQs only. On the contrary, ChatGPT scored a total of 140 marks out of 200. Among different types of questions, ChatGPT showed its best performance in answering information-based MCQs followed by logical reasoning types of questions (Table 2, Figure 3).

**Table 2:** Descriptive analysis of students' and ChatGPT's performance SD, standard deviation; IQR, inter quartile range; n=100

		Clinical questions	Logical reasoning	Comprehensive	Ethical reasoning	Information-based MCQ	MCQ logical reasoning	Total
	Max marks	25	32	95	8	25	15	200
Students’ performance (n=100)	Mean±SD	11.7±4.21	11.5±5.43	42.1±14.2	4.17±1.07	14.9±4.18	12.0±2.94	96.4±26.5
	Standard error mean	0.421	0.543	1.42	0.107	0.418	0.294	2.65
	Median	11.8	11.3	42.0	4.50	15.0	13.0	94.0
	IQR	5.13	7.5	20.1	1.63	5.25	3.00	35.5
	Range	3-23.5	1-26.5	8-75	1.5-6	2-22	4-18	27.5-164
ChatGPT's marks		14 (56%)	25.5 (80%)	61 (64%)	5.5 (69%)	23 (92%)	11 (73%)	140

We have compared the overall performance of 100 students with ChatGPT. Assuming ChatGPT had similar performance thrice, on the application of unpaired t-test, the difference was statistically significant. ChatGPT was well ahead of students in overall marks comparison (96.5±26.5 vs. 140±19.9, p=0.0058, Table 3).

**Table 3:** Comparison of students’ vs. ChatGPT’s performance SD, standard deviation; p<0.05 is statistically significant

	Mean±SD	Degree of freedom (d.f.)	t-score	p-value (two-tailed)
Students	96.5±26.5	101	43.5	0.0058
ChatGPT	140±19.9			

We also analyzed the time taken by ChatGPT to respond to the MCQs. For this, the MCQs were divided into two groups: information-based (n=25) and logical reasoning-based (n=15). ChatGPT took 2.02 seconds on average (SD=0.477, median: 1.87 seconds) to answer the information-based MCQs. In the case of logical reasoning-based MCQs, it took 3.10 seconds on average (SD=0.882, median: 2.95 seconds). Unpaired t-test showed that the average time taken for logical reasoning type of MCQs was higher (p<0.005) than the average time taken for information-based MCQs, and the difference was statistically significant (Table 4).

**Table 4:** Comparison of time taken by ChatGPT in answering two groups of MCQs SD, standard deviation, p<0.05 is statistically significant

Time taken by ChatGPT (seconds per question)	MCQs information-based (n=25)	MCQ logical reasoning (n=15)
Mean±SD	2.02±0.477	3.10±0.882
Standard error mean	0.953	0.228
Degree of freedom (d.f.)	38	
p-value	0.000	
Median	1.87	2.95
Range	1.34-3.35	1.79-5.01
All 100 students took 30 minutes to attempt 40 MCQ-based questions (45 seconds per question)		

**Figure 4:**
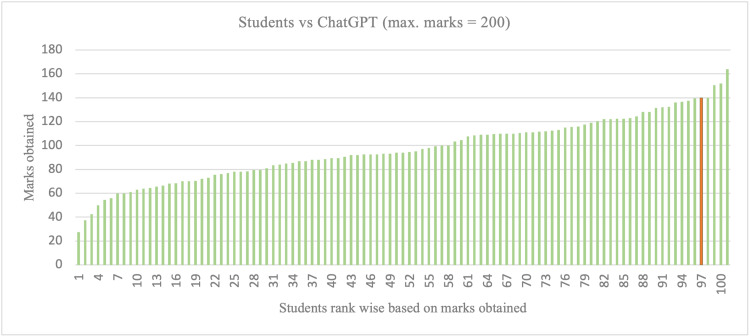
Students vs. ChatGPT - standing out of the crowd (roll numbers of the students were randomly assigned to hide their identity and any untoward correlation) The red color bar denotes the marks/position of ChatGPT

Based on these results, it can be concluded that ChatGPT demonstrated successful performance in the exam. Figure 4 shows an example of how it performed compared to the 100 students.

**Figure 5:**
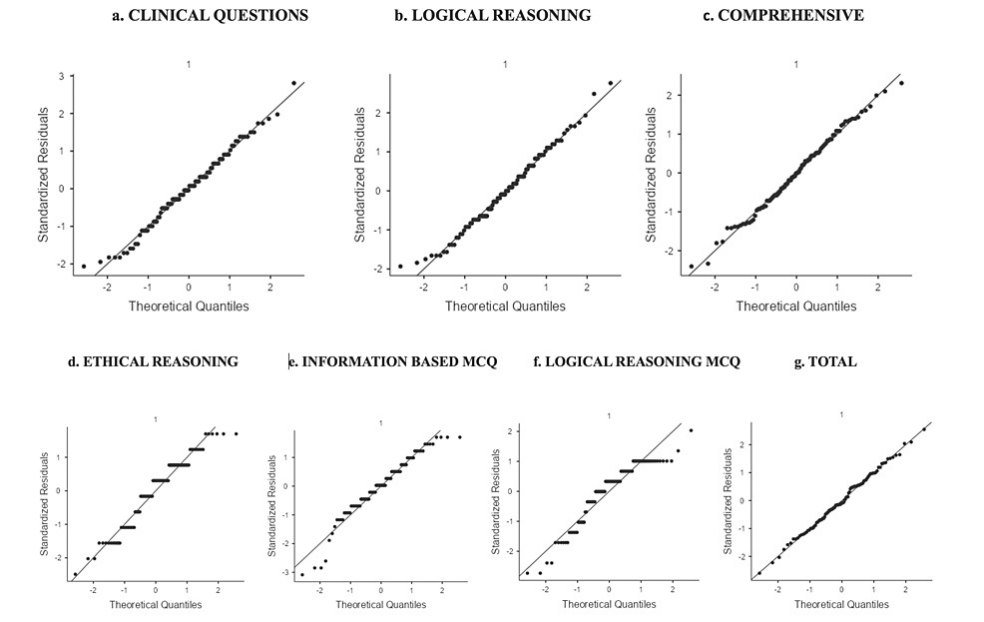
Q-Q plots of marks distribution (a) to (g) shows the Q-Q plots for different sets of questions

Figure 5 shows the Q-Q plots of mark distribution in different sets of questions. It shows the near-homogeneous distribution of data.

## Discussion

This study aimed to test and compare the performance of ChatGPT in answering questions from medical biochemistry in a real test scenario and analyze the former’s strengths and weaknesses. Our findings showed that ChatGPT performed well, obtained 70% marks, and ranked fifth among the class. The AI chatbot obtained higher marks than the mean marks of the students in almost all types of question sets except in MCQs based on logical reasoning (Table 2, Table 3, and Figure 4). This result demonstrates the potential power of the chat generative pre-trained transformer represented by ChatGPT to generate constructive, reasoning-based answers in the medical field. Several investigators have also evaluated the potential of ChatGPT in medical education. An Italian study by Bonetti et al. reported that ChatGPT’s score was ranked in the top 98.8th percentile among more than 15000 graduates appearing for the Italian Residency Admission National Exam [13]. According to Kung et al., ChatGPT demonstrated a high level of concordance and insight in its explanations while answering the questions of the United States Medical Licensing Exam (USMLE). It performed at or near the passing threshold for all three steps without any training [8]. In another study, Gilson et al. found that the same AI chatbot easily scored passing marks for a third-year medical student in the USMLE examination [14]. Another Turkey-based study by Talan et al. demonstrated that ChatGPT's performance was better than the average performance of the students enrolled in the anatomy course at the Faculty of Health Sciences, Turkey [15]. The same AI chatbot also performed well in a family medicine examination held at Antwerp University, Belgium [16]. Friederichs et al. showed that in the licensing examination of Germany, ChatGPT performed well and outperformed almost all medical students in years one to three [9].

Few Indian medical educators and researchers also have explored the capability of ChatGPT. Studies by Banerjee et al., Ghosh et al., Sinha et al., and Das et al. in the subjects of physiology, biochemistry, pathology, and microbiology, respectively, explored the possible potential of the AI chatbot to be a successful tool for answering questions requiring higher-order thinking in these subjects [17-20]. The observations of our study are similar to these studies and establish the fact that AI chatbots can answer in a human-like way, which requires rational thinking. Few studies, though, had some different observations and disagreed with our findings. A study from Korea by Huh showed that ChatGPT performed poorly in medical parasitology examinations compared to medical students [21]. Another study by Weng et al. showed that ChatGPT failed Taiwan’s family medicine board exam [22]. There were limitations in both these studies as stated by the authors [21,22]. Removal of these limitations could have altered the outcome.

In our study, ChatGPT scored comparatively lesser marks in clinical questions (Table 2), which requires an ability to connect different physiological and pathological facts and generate an answer with higher-order interpretative ability. This could be because ChatGPT cannot still construct a real-life clinical scenario and correlate different facts related to medical knowledge, which are available as plain text in its database. This hypothesis is supported by the marks obtained by the same AI chatbot in logical reasoning and different sets of MCQs, where it performed well (Table 2). It shows that ChatGPT knows different sub-topics with a comprehensive understanding and higher-order thinking, and it has problem-solving abilities in the subject matter of medical biochemistry equivalent to a first professional MBBS student.

On analyzing the ChatGPT’s ability to answer different types of MCQs and comparing the time taken to answer them, it was found that simple recall or information-based MCQs took less time, which was statistically significant when compared to logical reasoning-based MCQs (Table 4). This can be explained by the fact that in the case of logical reasoning, the chatbot had to process all the text-based information in its deep learning model to generate a suitable answer, which had to match with one of the four options provided in the specific MCQ.

**Figure 6:**
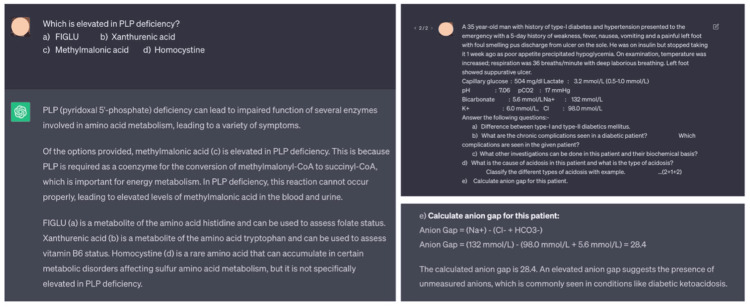
Screenshots of ChatGPT’s wrong answer to questions The registered user profile image has been blurred to prevent identification and maintain anonymity during peer review.

A few observations of this study are worth mentioning and need to be described. The descriptive answers generated by the ChatGPT had some quality issues. Many times the answers were monotonous and lacked proper structuring. The answers did not have any flow chart or line diagram. Though sufficient information was presented in an answer, they were poorly organized. At the end of every descriptive answer, a paragraph of the answer summary was generated, which was unnecessary. During the whole process of question and answering, ChatGPT never replied as "don’t know." In case of the wrong answers, the chatbot tried to establish its answer with irrelevant references. Not only that, the chatbot had performed poorly in a question that involved mathematical formulas and calculations (Figure 6). All these patterns could be due to its algorithm structure and the inherent behavior of the LLM [9].

The release of the AI Chatbot has raised concerns among medical teachers and institutions, about whether ChatGPT will be able to replace formal teaching and hamper the role of assignments in medical teaching. It is a fact that ChatGPT does not pose any threat to the assessment process, which is taken face to face. However, with the availability of chatbots like ChatGPT, online submission of assessments will not be fully appropriate. Students can easily write an answer to a question generated by a chatbot and claim it as his/her own. Though there are various tools available based on machine learning that can detect AI-generated text, those tools are not always accurate, and misclassification can happen, which can lead to controversies [2]. Moreover, this malpractice can prevent a student from thinking rationally and applying his/her knowledge to solve a higher-order question. 

Another point of concern in using ChatGPT as a learning tool for medical students is the validity or correctness of the response generated by it. It is necessary to ensure that the AI chatbots are designed in such a way that they can generate validated responses and authentic references for medical students.

Considering all the negative impacts of ChatGPT discussed above, we still cannot overlook the benefits that the AI chatbot can potentially offer in improving medical education. If the students are trained to use these AIs optimally, they can easily search for an answer and the reasoning behind it while studying. The answer generated by the chatbots may not be available on simple internet searches, as upon searching the internet with a specific question, it always produces the relevant information in a disorganized manner, from where the exact answer needs to be extracted. ChatGPT like chatbots always produces linguistically well-structured, high-quality answers that provide a personalized learning experience to the students.

**Figure 7:**
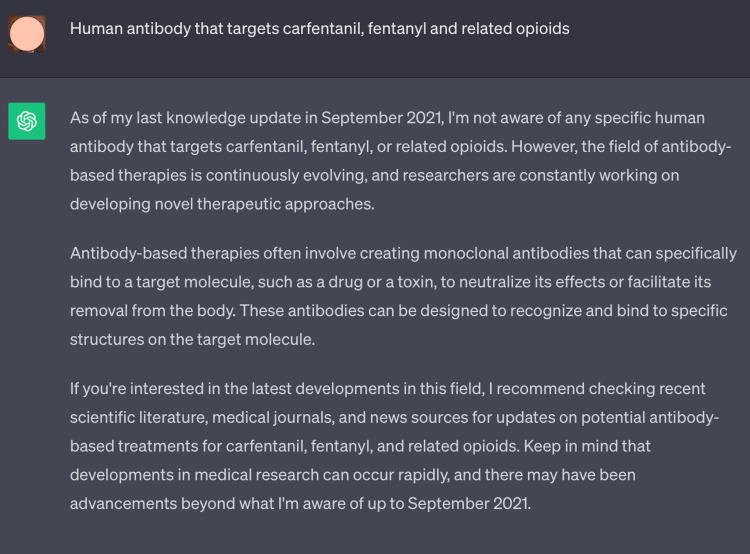
Screenshot of ChatGPT’s response upon asking questions based on recent advances in biochemistry that occurred after 2021 The registered user profile image has been blurred to prevent identification and maintain anonymity during peer review.

**Figure 8:**
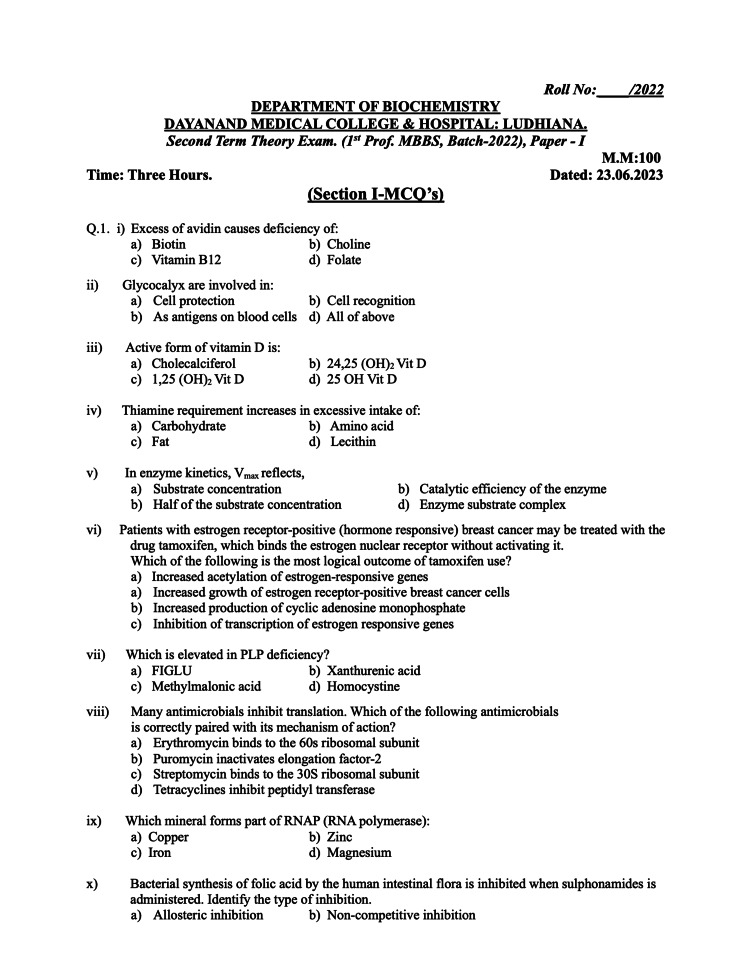
Second term theory exam (first professional MBBS, Batch 2022), paper - I, page 1

**Figure 9:**
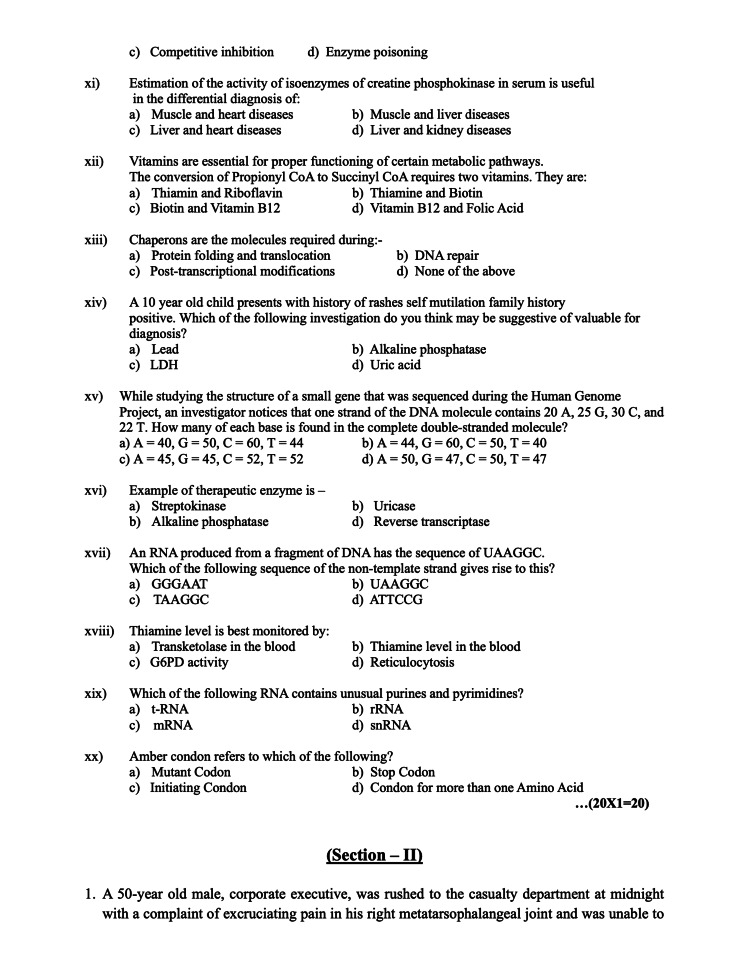
Second term theory exam (first professional MBBS, Batch 2022), paper - I, page 2

**Figure 10:**
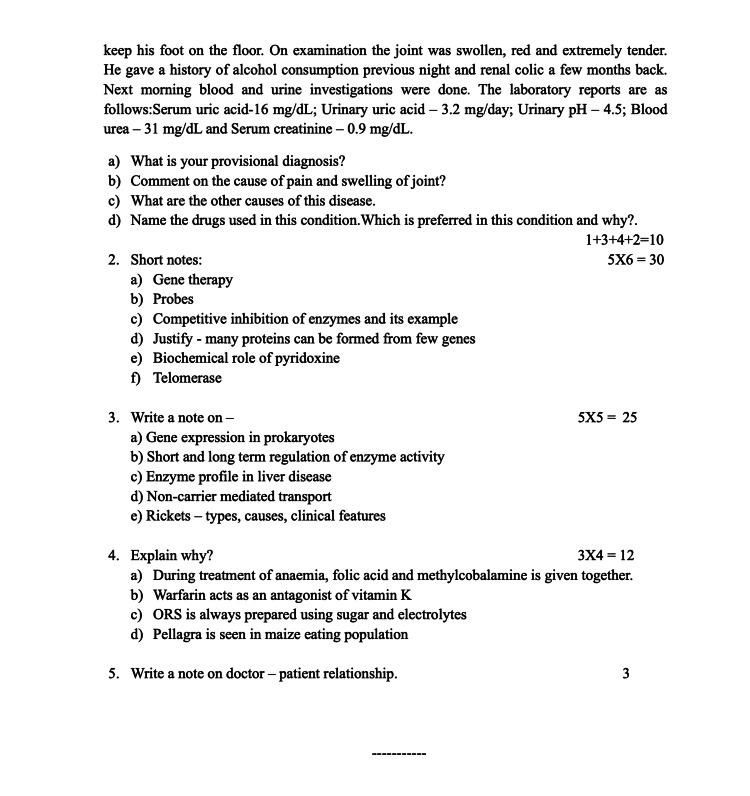
Second term theory exam (first professional MBBS, Batch 2022), paper - I, page 3

**Figure 11:**
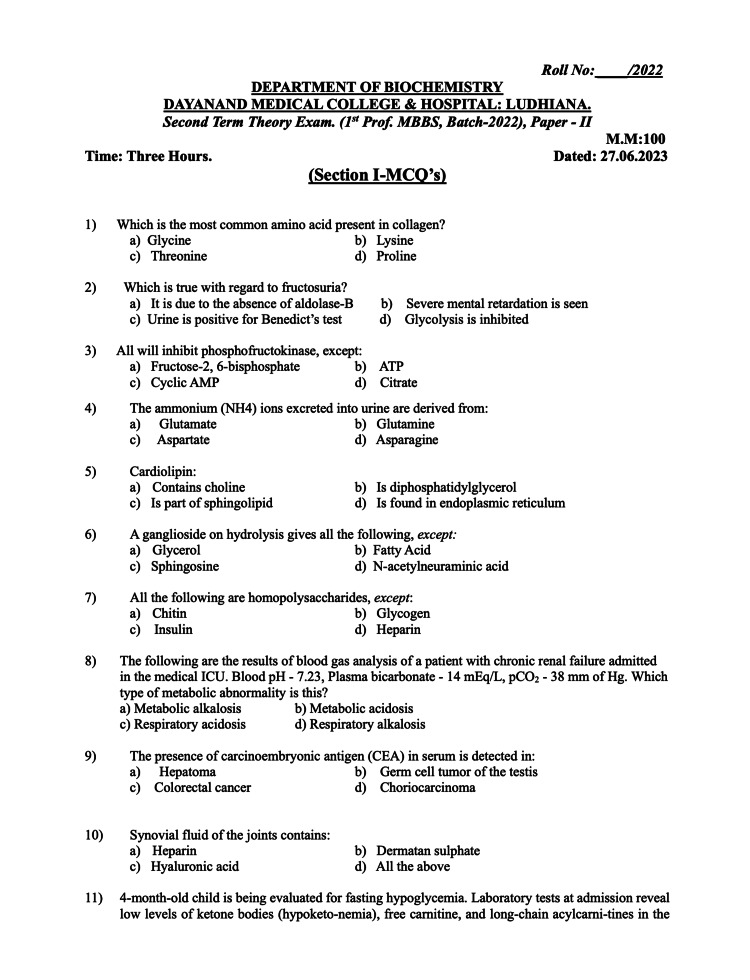
Second term theory exam (first professional MBBS, Batch 2022), paper - II, page 1

**Figure 12:**
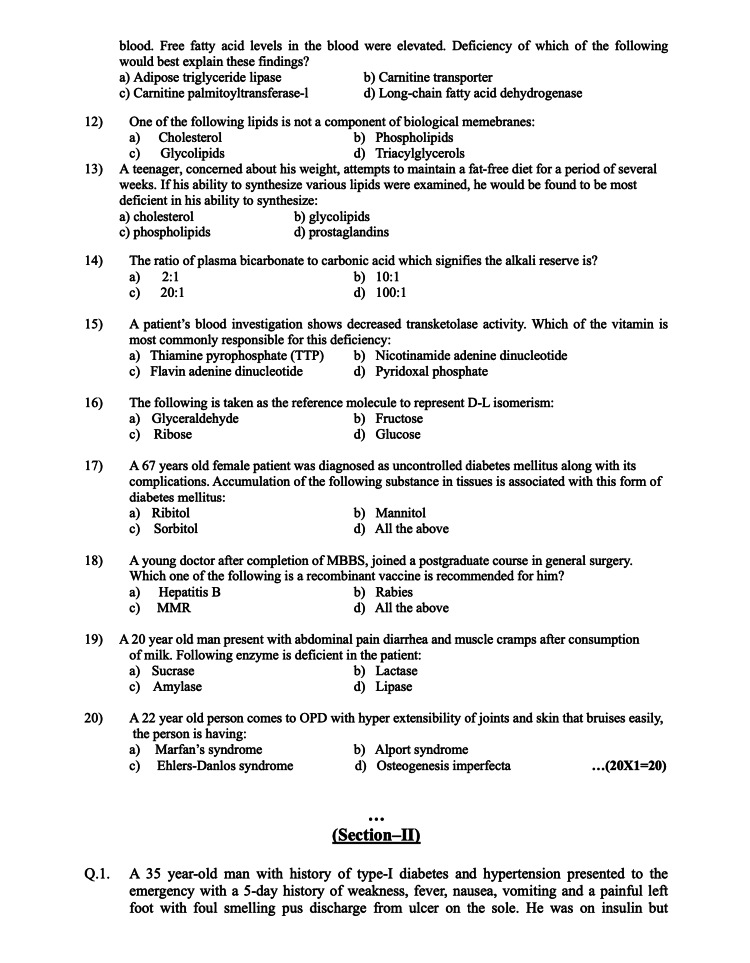
Second term theory exam (first professional MBBS, Batch 2022), paper - II, page 2

**Figure 13:**
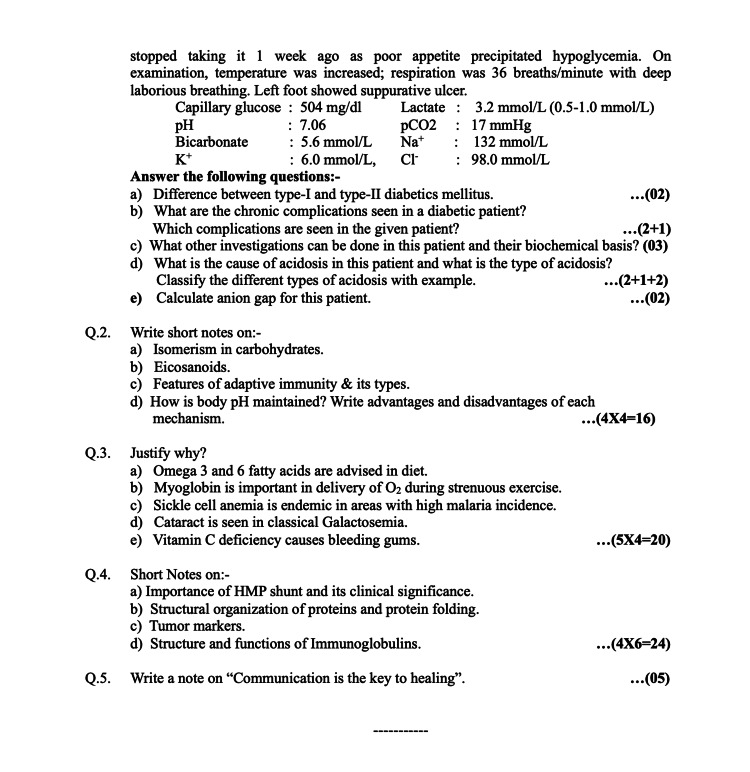
Second term theory exam (first professional MBBS, Batch 2022), paper - II, page 3

It is a known fact that GPT 3.5 is equipped with data till September 2021 and the latest development in the medical field is inaccessible to it. We tried to demonstrate this fact by asking a question regarding a recent topic of clinical biochemistry (targeted antibody) and ChatGPT expressed its inability to answer the question (Figure 7).

As the medical field is continuously evolving and newer information is constantly coming in, AI must update itself to provide accurate health-related information, which is of importance for medical students as well as practitioners. 

To explore the full potential of these AI chatbots based on the LLM, a potential follow-up study is necessary which could compare the performance of the updated models with our findings and see to what extent these AI platforms can progress in learning medical factual knowledge. 

Limitations

As GPT 3.5 does not possess the ability to process images, we had to a exclude few image/diagram-based questions. As the study was based on questions from biochemistry block I and block II of the first professional MBBS, a few topics of recent advances were left out, the inclusion of which could have altered the marks obtained by the Chatbot up to some extent. The study was designed as an everyday approach to AI and was not designed to show the maximum performance of the model. Thus, the validity of technical response behavior is limited. It was also unclear whether ChatGPT had already been tested on these questions or similar questions and whether the model had learned from those inputs.

## Conclusions

ChatGPT’s performance in our study was surprising. The chatbot performed well in both descriptive questions and MCQs and outperformed almost all the students in the class. It showed that the chatbot is not only good at choosing answers from a given set of options in MCQs but can also construct a sufficiently well-versed, validated descriptive answer for medical biochemistry questions. As we have used GPT 3.5 for our study, a few quality issues in answers given by ChatGPT were also identified, which we believe can be overcome by using GPT4. Medical educators must be aware of the strengths and weaknesses of these generative AI and guide their students to use these AI chatbots ethically in their academic setting. We firmly believe that as LLMs like ChatGPT are getting refined every day by their users, it will soon achieve a certain maturity level to start impacting modern medicine education and practice on a large scale.

## References

[1] Howard J (2019). Artificial intelligence: implications for the future of work. Am J Ind Med.

[2] Ali K, Barhom N, Tamimi F, Duggal M (2023). ChatGPT—A double-edged sword for healthcare education? Implications for assessments of dental students. Eur J Dent Educ.

[3] King MR (2023). The future of AI in medicine: a perspective from a chatbot. Ann Biomed Eng.

[4] Girimonte D, Izzo D (2007). Artificial intelligence for space applications. Intelligent Computing Everywhere.

[5] Sharma GD, Yadav A, Chopra R (2020). Artificial intelligence and effective governance: a review, critique and research agenda. Sustain Futures.

[6] (2023). What is ChatGPT: The History of ChatGPT - Open AI (2023). https://digitalscholar.in/history-of-chatgpt/..

[7] Vázquez-Cano E, Ramírez-Hurtado JM, Sáez-López JM, López-Meneses E (2023). ChatGPT: the brightest student in the class. Think Skills Creat.

[8] Kung TH, Cheatham M, Medenilla A (2023). Performance of ChatGPT on USMLE: potential for AI-assisted medical education using large language models. PLOS Digit Health.

[9] Friederichs H, Friederichs WJ, März M (2023). ChatGPT in medical school: how successful is AI in progress testing?. Med Educ Online.

[10] (2023). Competency Based Undergraduate Programme: National Medical Commission, India. https://www.nmc.org.in/information-desk/for-colleges/ug-curriculum/.

[11] (2023). ChatGPT Statistics & Facts. https://seo.ai/blog/chatgpt-user-statistics-facts.

[12] (2023). The jamovi project. https://www.jamovi.org..

[13] Alessandri Bonetti M, Giorgino R, Gallo Afflitto G, De Lorenzi F, Egro FM (2023). How does ChatGPT perform on the Italian residency admission national exam compared to 15,869 medical graduates?. Ann Biomed Eng.

[14] Gilson A, Safranek CW, Huang T, Socrates V, Chi L, Taylor RA, Chartash D (2023). How does ChatGPT perform on the United States Medical Licensing Examination? The implications of large language models for medical education and knowledge assessment. JMIR Med Educ.

[15] Talan T, Kalinkara Y (2023). The role of artificial intelligence in higher education: ChatGPT assessment for anatomy course. Uluslararası Yönetim Bilişim Sistemleri ve Bilgisayar Bilimleri Dergisi.

[16] Morreel S, Mathysen D, Verhoeven V (2023). Aye, AI! ChatGPT passes multiple-choice family medicine exam. Med Teach.

[17] Banerjee A, Ahmad A, Bhalla P, Goyal K (2023). Assessing the efficacy of ChatGPT in solving questions based on the core concepts in physiology. Cureus.

[18] Ghosh A, Bir A (2023). Evaluating ChatGPT’s ability to solve higher-order questions on the competency-based medical education curriculum in medical biochemistry. Cureus.

[19] Sinha RK, Deb Roy A, Kumar N, Mondal H (2023). Applicability of ChatGPT in assisting to solve higher order problems in pathology. Cureus.

[20] Das D, Kumar N, Longjam LA, Sinha R, Deb Roy A, Mondal H, Gupta P (2023). Assessing the capability of ChatGPT in answering first- and second-order knowledge questions on microbiology as per competency-based medical education curriculum. Cureus.

[21] Huh S (2023). Are ChatGPT’s knowledge and interpretation ability comparable to those of medical students in Korea for taking a parasitology examination?: a descriptive study. J Educ Eval Health Prof.

[22] Weng TL, Wang YM, Chang S, Chen TJ, Hwang SJ (2023). ChatGPT failed Taiwan’s Family Medicine Board Exam. J Chin Med Assoc.

